# Sensitive and selective determination of vitamin B2 in non-alcoholic beverage and milk samples at poly (glutamic acid)/zinc oxide nanoparticles modified carbon paste electrode

**DOI:** 10.1186/s13065-022-00863-5

**Published:** 2022-09-18

**Authors:** Gizaw Tesfaye, Negussie Negash, Merid Tessema

**Affiliations:** Department of Chemistry, College of Natural and Computational Sciences, Addis Ababa University, P. O. Box 1176, Addis Ababa, Ethiopia

**Keywords:** Milk, Non-alcoholic beverage, Poly (glutamic acid), ZnO NPs, Vitamin B2

## Abstract

**Background:**

The deficiency of vitamin B2 can lead to many health problems. Therefore, it is necessary to develop a sensitive, selective and fast method for the determination of vitamin B2 in food samples. In this work, a sensitive, selective and low-cost electrochemical sensor was developed using poly (glutamic acid) and Zinc oxide nanoparticles (ZnO NPs) for vitamin B2 in non-alcoholic beverage and milk samples.

**Methods:**

The modification of the electrode surface was carried out by electropolymerization of glutamic acid on ZnO NPs–carbon paste electrode (ZnO NPS–CPE). The prepared electrodes were characterized by cyclic voltammetry (CV), electrochemical impedance spectroscopy (EIS), scanning electron microscopy (SEM) and X-Ray diffraction (XRD). CV and square wave voltammetry (SWV) were used to investigate the electrochemical behavior of vitamin B2 at the modified electrode. The effect of various parameters such as amount of ZnO NPs, polymerization cycle, concentration of the monomer, pH, scan rate and accumulation time were optimized to obtain maximum sensitivity at the modified electrode.

**Results:**

The developed sensor showed high electrocatalytic activity towards vitamin B2. Under the optimized conditions, the developed sensor showed a linear response in the range 0.005–10 µM with a low detection limit of (LOD) 0.0007 ± 0.00001 µM and high sensitivity of 21.53 µA/µM.

**Conclusions:**

A reproducible, repeatable, stable and selective sensor was successfully applied for the quantification of vitamin B2 in beverage and milk samples with acceptable recoveries in the range of 88–101%.

**Supplementary Information:**

The online version contains supplementary material available at 10.1186/s13065-022-00863-5.

## Background

**Fig. 1:**
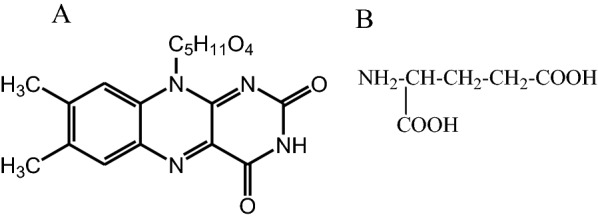
Chemical structures of **A** Vitamin B2 and **B** glutamic acid

Vitamins are an important group of organic compounds that are necessary for normal cell growth and regeneration where their absence can lead to many diseases [1–3]. Riboflavin or Vitamin B_2_ (Fig. 1A) is a water-soluble vitamin and an essential component of flavoenzymes the body uses to catalyze carbohydrates, protein and fat to produce energy [4–7]. Furthermore, it promotes cell growth and regeneration [8]. It cannot be synthesized in the human body and therefore must be obtained from dietary sources such as vegetables, fruit, drug, liver, cheese, milk, meat and eggs [6, 9, 12]. The Recommended Dietary Allowance of riboflavin ranges from 1.0 to 1.3 mg/d for adults and even higher amounts are advised to pregnant and lactating women [7, 13, 14]. The deficiency of vitamin B2 causes eye lesions, skin disorder, anemia, night blindness, sore throat, loss of hair, swollen tongue and impaired nerve functions [1, 7, 9, 10, 14, 15]. However, taking a high dose of Vitamin B_2_ leads to oxidative damage to DNA and liver in human bodies [7, 11].

Therefore, it is necessary to develop a sensitive, selective and fast method for the determination of vitamin B2 in food samples. Various analytical methods have been developed for the identification and quantification of vitamin B2 such as spectrophotometry [16, 17], high performance liquid chromatography [18], capillary electrophoresis [19, 20], liquid chromatography-mass spectrometry [21], fluorimetry [22] and liquid chromatography-tandem mass spectrometry [23]. Although these methods provide high accuracy and low detection limits, they require time-consuming procedures, expensive reagents and sophisticated measuring equipment [6, 8, 9, 24, 25].

Electroanalytical methods have been developed for the determination of many compounds in biological and environmental samples. Compared to chromatography and spectroscopy, electroanalytical methods have several advantages, such as low-cost, high sensitivity, easy operation and quick response towards the determination of analytes of interest [8, 24, 27]. Furthermore, electroanalytical methods provide information about reaction mechanisms and kinetics that occur at the electrode surface [28]. Since vitamin B2 is an electroactive molecule, it can easily be determined by electrochemical techniques. However, the electrochemical determinations of the target analyte at bare electrode have a number of drawbacks, such as low sensitivity, poor selectivity, slow electron transfer kinetics, high overpotential and electrode fouling [8]. These drawbacks can be overcome by modifying the electrode surface.

CPE has been widely used as a working electrode for the determination of biologically active molecules due to renewable surface, low background current, wide potential window, low ohmic resistance, low cost and the ease to incorporate various modifiers during paste preparation [7].

The key point in the construction of an electrochemical sensor is the selection of an appropriate modifier to increase the sensitivity, achieve lower detection limit, prevent electrode fouling and increase selectivity of the electrochemical response. Recently, Polymers modified carbon paste electrodes prepared by electropolymerization have been widely used for the electrochemical determination of different analytes because the modified electrodes exhibited good stability, high sensitivity, reproducibility and selectivity [29]. Compared to other polymers, poly (amino acids) prepared by electropolymerization of amino acids have several advantages such as easy processibilty in aqueous solution which minimizes the use of toxic solvents, availability of various functional groups such as carboxylic (COOH), amino (NH_2_) and hydroxyl (OH) for effective interaction with the target analyte, high stability and low-cost [9, 30, 32]. Recently, different poly (amino acids) have been reported for the electrochemical determination of various analytes [7–9, 12, 26, 28, 29, 31, 33, 34]. Glutamic acid (Fig. 1B) is among the common amino acids that can easily be electropolymerized on the electrode surface to form poly (glutamic acid) [35]. Poly (glutamic acid) contains glutamate repeating units and free protonated carboxylic groups (pKa = 4.45) by linking α-amino and δ-carboxylic acid functional groups, which show enhanced properties in electrochemical applications [36]. Electrodes modified with poly (glutamic acid) have been successfully used in the determination of various analytes such as Epinephrine [37], Hydrazine [38], L-tryptophan [35], Bisphenol A [36], Rutin [39] and Gallic acid [40]. The modified electrodes showed high electrocatalytic activity and stability toward the target analytes.

On the other hand, different nanomaterials modified electrodes have been widely used in electrochemical sensors and biosensors owing to their high surface area, good electrical conductivity, high chemical stability and excellent catalytic activity. Among the different nanomaterials, metal oxide nanomaterials have received much attention due to their high catalytic efficiency, strong adsorption ability, biocompatibility, chemical and biological stability, low toxicity and low cost for large-scale production [41, 42]. ZnO is an n-type semiconductor metal oxide with a wide bandgap of 3.37 eV and a large exciton binding energy of 60 meV [43]. ZnO NPs were widely used for electrode modification because of their high surface area, high isoelectric point, easy availability, high catalytic ability and biocompatibility [27, 44, 45].

The synergic effect of the combined use of metal oxide nanoparticles and polymers has been explored in recent years for the electrochemical determination of various compounds. Metal oxide nanoparticles and polymers composite improve the electrochemical performance of bare electrodes, providing high electroactive surface area and, thereby, higher sensitivities and lower detection limits.

**Scheme 1:**
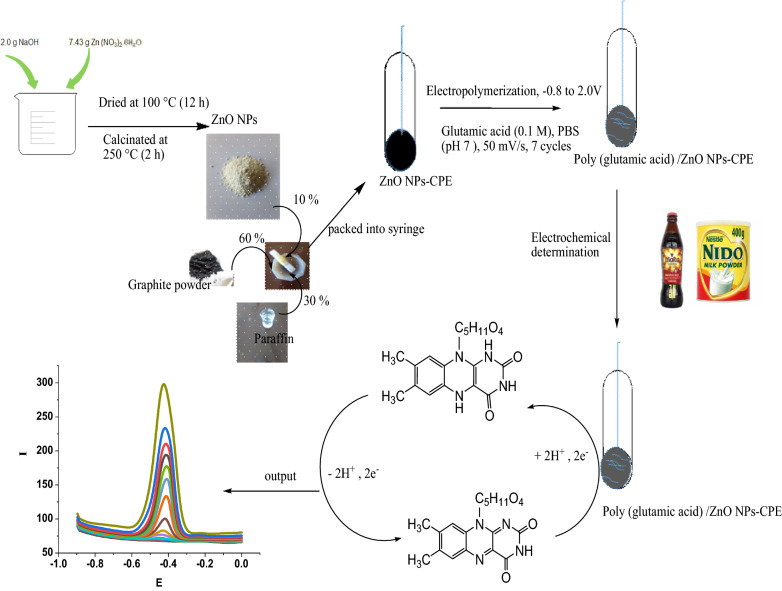
Procedure for the preparation of Poly (glutamic acid)/ZnO NPs–CPE and mechanism for the detection of vitamin B2

In this work, considering the combined advantages of polymer and metal oxide nanoparticles, we have fabricated poly (glutamic acid) and ZnO NPs composite modified CPE for the electrochemical determination of vitamin B2. The procedure for the Poly (glutamic acid)/ZnO NPs–CPE preparation and sensing mechanism is shown in Scheme 1. The modified electrode was characterized by CV, EIS, SEM and XRD. The electrochemical behavior of vitamin B2 was studied using CV and SWV. Finally, the proposed method was applied for the determination of vitamin B2 in milk and non-alcoholic beverage samples.

## Experimental

### Reagents and chemicals

All chemicals used in this study were analytical grade and were used without any further purification: Riboflavin (≥ 98%), glutamic acid (99%), zinc nitrate (> 96%), acetic acid (99.8%), glucose (≥ 99.5%), starch (A.R. grade), potassium ferrichexacyanide (99%), Sucrose (≥ 99.5%), lactose (≥ 99%), vitamin B12 (≥ 98%), vitamin B9 (≥ 97%), sodium hydroxide (98%) and hydrochloric acid (37%) were obtained from Sigma-Aldrich (USA). Graphite powder (particle size < 50 µm, 99.5% pure) and paraffin oil (binder, 98%) for the preparation of carbon paste electrodes were purchased from Merck (Darmstadt, Germany). Citric acid (99%), sodium citrate (98%) and dipotassium hydrogen phosphate (98%) were obtained from Research–Lab. Chem. Industries (Mumbai, India). Potassium nitrate (99%), sodium sulfate (≥ 99%)_,_ ascorbic acid (≥ 99.7%), vitamin B6 (≥ 98%) and vitamin B1 (≥ 99%) were obtained from (BDH, England). Potassium dihydrogen phosphate (98%), magnesium chloride (99.5%), copper nitrate (98%), iron (II) nitrate (98%), sodium carbonate (99.5%) and sodium bicarbonate (99.7%) were obtained from Hopkin and Williams LTD (England). Potassium chloride (98%) was purchased from Riedel-de Haën-Franc. Boric acid (99.5%) was obtained from Carlo erba reagent SPA (Italy).

1 mM stock solution of riboflavin was prepared by dissolving accurately weighed amounts of riboflavin in a minimum amount of 0.1 M sodium hydroxide and diluting it with distilled water. The stock solution was stored at 4 ^o^ C in a refrigerator until use. 0.1 M glutamic acid solution was prepared by dissolving the appropriate amount of glutamic acid in PBS pH 7. Phosphate buffer solutions were prepared by mixing 0.1 M potassium dihydrogen phosphate and 0.1 M dipotassium hydrogen phosphate. The pH was adjusted using 0.1 M NaOH and 0.1 M HCl solution. The working solutions were prepared by diluting the stock solutions with phosphate buffer solution pH 6.

### Apparatus and instruments

Electrochemical measurement was conducted on CHI 760D Electrochemical Workstation (CH Instruments, USA) consisting of bare CPE or modified CPE as a working electrode, platinum wire as counter electrode and Ag/AgCl (3 M KCl) as a reference electrode. An electronic digital balance (Model: Scientech: ZSA 120) and pH meter (sensION, SHA Snilu Instruments Co. Ltd., China) were used for weighing chemicals and measuring the pH of solutions, respectively. Scanning electron microscopy (Cx-200 coxem, Korea) was used to determine the morphology of bare carbon paste and modified carbon paste electrodes. The crystal structure and purity of the synthesized ZnO NPs were determined by an X-ray diffractometer (XRD, 38066 Riva, d/G. ViaM. Misone, 11/D (TN), Italy).

## Real sample preparation

### Non-alcoholic beverage sample

Malt guinness samples (a non-alcoholic beverage, Meta Abo Brewery S. C) were purchased from a local grocery in Addis Ababa, Ethiopia and stored in a refrigerator prior to analysis. Before voltammetric determination, the liquid sample was transferred into a beaker and degassed in an ultrasonic bath. Then, 1.0 ml of the sample was diluted to 10 ml with 0.10 M PBS pH 6 solution**.** For the recovery test, the samples were spiked with a stock solution of vitamin B2.

### Milk sample

Milk powder samples (Nido Fortified Milk Powder, Netherlands) were purchased from a local supermarket in Addis Ababa, Ethiopia and stored in a refrigerator prior to analysis. For the electrochemical determination of vitamin B2, the milk sample was treated according to the procedure described in the literature [46]. Briefly, 5 g of the milk powder was dissolved in hot distilled water and glacial acetic acid was added dropwise to precipitate out proteins. Then, the resulting solution was digested on a water bath for 20 min and centrifuged for 10 min. Finally, a clear filtrate was obtained after filtering the supernatant using Whatman filter paper. For the SWV determination of Vitamin B2 by standard addition method, l ml of the filtrate was diluted to 10 ml with 0.1 M PBS pH 6.0. For the recovery test, the samples were spiked with various concentrations of the standard solution of Vitamin B2.

## Synthesis of nanomaterials and electrode modification

### Preparation of ZnO NPs

The ZnO NPs were synthesized according to the procedure described in the literature [47]. Briefly, 2.0 g sodium hydroxide was dissolved in 100 ml of deionized water and heated to 55 °C with constant stirring. After achieving to this temperature, a solution of 7.43 g of Zn (NO_3_)_2_.6H_2_O in 100 ml of deionized water was added slowly into the NaOH aqueous solution under continuous stirring. The resulting solution was filtered, and then the precipitate was washed with deionized water and ethanol several times and was dried at 100 °C for 12 h. The dried precipitate was calcined at 250 °C for 2 h to obtain a nano-sized ZnO.

### Preparation of bare and modified carbon paste electrode

Bare carbon paste electrode (BCPE) was prepared by mixing 70% w/w graphite powder and 30% w/w paraffin and homogenizing it for 25 min using mortar and pestle. The paste was then after packed into a syringe and electrical contact was provided using a copper wire. To prepare ZnO NPs–CPE, 60% graphite powder, 10% ZnO NPs, and 30% paraffin oil were mixed and homogenized using mortar and pestle. The poly (glutamic acid) film-modified ZnO NPs–CPE was prepared by cyclic voltammetry by cycling the potential in the potential range −  0.8–2.0 V at a scan rate of 50 mV/s in 0.1 M glutamic acid in 0.1 M PBS pH 7.0 for 7 cycles. The obtained modified electrode Poly (glutamic acid)/ZnO NPs–CPE was washed with double distilled water to remove unreacted glutamic acid. For comparison purposes, Poly (glutamic acid) modified CPE was also prepared with the same procedures described above.

## Results and discussions

### Electropolymerization of glutamic acid on ZnO NPs–CPE

**Fig. 2:**
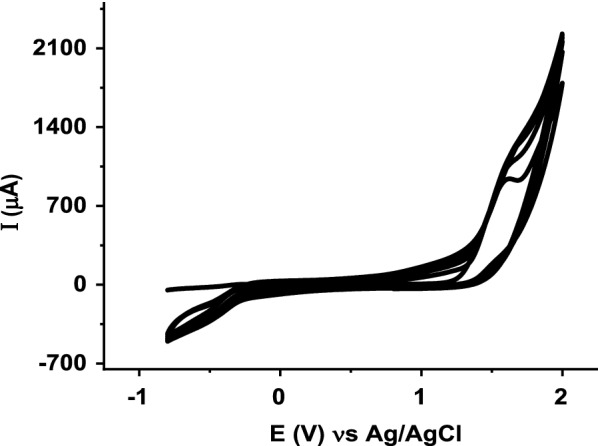
Cyclic Voltammogram (CVs) of the electropolymerization of 0.1 M glutamic acid in 0.1 M PBS (pH 7) at ZnO–CPE in the potential range − 0.8 and 2.0 V for 7 cycles at scan rate of 50 mV s^−1^

**Scheme 2:**
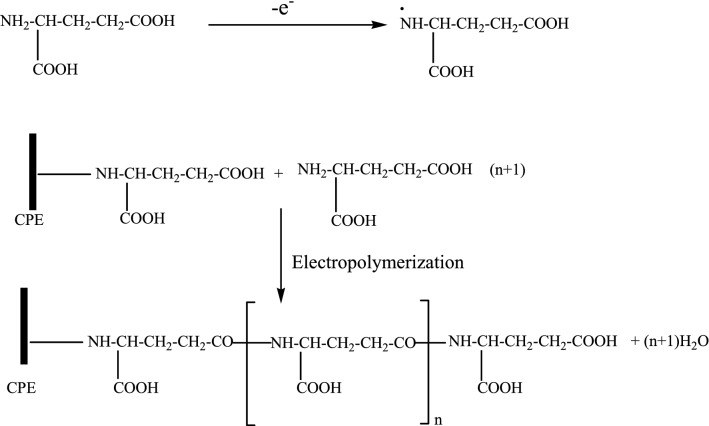
Mechanism of electropolymerization of glutamic acid on ZnO NPs–CPE

Electrochemical polymerization of glutamic acid on the ZnO NPs–CPE was carried out with cyclic voltammetry in the potential range − 0.8 to 2.0 V at a scan rate of 50 mV s^−1^ in 0.1 M PBS solution (pH 7) containing 0.1 M glutamic acid. Figure 2 shows the CV curves for the electropolymerization of glutamic acid on the ZnO NPs–CPE. During the polymerization process, an anodic peak was obtained at 1.54 V corresponding to the formation of monomer radical which initiates the polymerization process. The oxidation peak current increased with the number of cycles, indicating the formation of a conductive polymer film on the ZnO NPs–CPE surface. According to the literature [36, 48], the glutamic acid monomer is oxidized first at a higher positive potential to form α- amino free radicals, and these cation radicals can form carbon–nitrogen links at the carbon electrode surface, and then after the poly (glutamic acid) films are formed as described in Scheme 2 [36, 48].

## Characterization of bare and modified carbon paste electrodes

### Electrochemical properties of bare and modified carbon paste electrodes

**Fig. 3:**
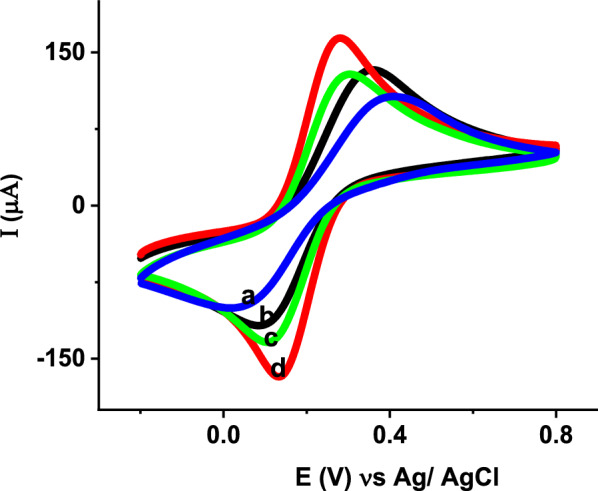
CVs of 5.0 mM [Fe (CN)_6_] ^3−/4−^ in 0.1 M KCl at **a** BCPE, **b** ZnO NPs–CPE, **c** poly (glutamic acid)/CPE and **d** poly (glutamic acid)/ZnO NPs–CPE at a scan rate of 100 mVs^−1^

The effects of modifying the CPE with ZnO NPs, poly (glutamic acid) and poly (glutamic acid)/ZnO NPs were investigated by CV and EIS. Figure 3 shows the CV responses of CPE (curve a), ZnO NPs–CPE (curve b), poly (glutamic acid) (curve c) and poly (glutamic acid)/ZnO NPs–CPE (curve d) in the presence of 5 mM [Fe(CN)_6_] ^3−/4−^. At the bare CPE weak redox peaks are obtained with peak—to—peak potential separation of 389 mV, indicating a slow electron transfer rate at bare CPE. For ZnO NPs–CPE the current response of [Fe(CN)_6_^3−/4−^] increased and the peak-to-peak potential separation decreased to 273 mV due to the high surface area and conductivity of ZnO NPs. Similarly, compared to the bare CPE, the redox peak currents increased and peak to peak potential separation decreased to 198 mV after electrodeposition of poly (glutamic acid) on the CPE due to high conductivity and more active sites provided by the polymer to allow more [Fe(CN)_6_^3−/4−^] to reach the electrode surface easily. Among all the modified electrodes, the highest current response and the lowest peak potential separation (147 mV) are obtained at poly (glutamic acid)/ZnO NPs–CPE due to the synergistic effect of ZnO NPs and poly (glutamic acid) for the electron transfer between [Fe(CN)_6_^3−/4−^] and poly(glutamic acid)/ZnO NPs–CPE. The electroactive surface areas of the bare CPE and modified CPEs were calculated using Randles–Sevcik Eq. (1) [7, 49].

1 $${\text{I}}_{{\text{p}}} = 2.{\text{69 }} \times {\text{ 1}}0^{5} {\text{AD}}^{{1/2}} {\text{n}}^{{3/2}} \upsilon ^{{1/2}} {\text{C}}$$

where I_p_ is the peak current (A), n is the number of electrons involved in the reaction (n = 1), A is the electrode active surface area (cm^2^), D is the diffusion coefficient of K_3_[Fe(CN)_6_] in 0.1 M KCl solution (7.6 × 10^–6^ cm^2^s^−1^), C is the concentration of K_3_[Fe(CN)_6_] (mol cm^−3^) and υ is the scan rate (V s^−1^). From the scan rate study, both the anodic and cathodic peak currents of all electrodes were proportional to the square root of the scan rate in the range 0.025 to 0.35 V/s. The regression equations obtained from CV analysis for the bare and modified electrodes are: Ip = 214.43 υ^1/2^ (Vs^−1^)^1/2^ + 34.6, Ip = 280.43 υ^1/2^ (Vs^−1^)^1/2^ + 39.22, Ip = 294.5 υ^1/2^ (Vs^−1^)^1/2^ + 50.58 and Ip = 334.94 υ^1/2^ (Vs^−1^)^1/2^ + 91.5 for CPE, ZnO NPs–CPE, poly (glutamic acid)/CPE and poly (glutamic acid)/ZnO NPs–CPE, respectively. Hence, from the slopes of the plot of Ip versus υ^1/2^ (Additional file 1: Fig. S1), the electroactive surface areas were calculated. According to the calculations, the electro-active surface areas are 0.058, 0.076, 0.079 and 0.093 cm^2^ for CPE, ZnO NPs–CPE, poly (glutamic acid)/CPE and poly (glutamic acid)/ZnO NPs–CPE, respectively. The increase in effective surface area after modification results in increased number of catalytic sites for electrochemical oxidation of vitamin B2.

**Fig. 4:**
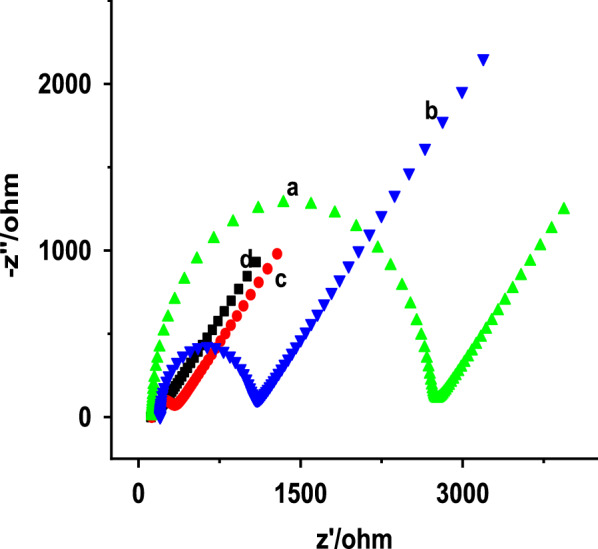
Nyquist plot of 5.0 mM [Fe(CN)_6_] ^3−/4−^ in 0.1 M KCl at **a** BCPE, **b** ZnO NPs–CPE, **c** Poly(glutamic acid)/CPE and **d** Poly(glutamic acid)/ZnO–CPE at frequency range of 0.01 Hz – 100 kHz, applied potential of 0.15 V and amplitude of 0.005 V

EIS is a very useful technique to analyze the electrochemical properties of bare and modified electrodes [11]. Figure 4 shows the Nyquist plots of EIS for the electrodes in 5.0 mM [Fe (CN)_6_] ^3−/4−^ redox probe containing 0.1 M KCl. The R_ct_ values obtained for the BCPE, ZnO NPs–CPE, poly (glutamic acid)/CPE and poly (glutamic acid)/ZnO NPs–CPE are 2568, 838.3, 185.3 and 52.03 Ω, respectively. It was clearly observed that the R_ct_ value of the BCPE was larger than that of ZnO NPs modified CPE, indicating that the ZnO NPs modified surface showed higher conductivity than bare CPE. Electrodeposition of poly (glutamic acid) on the surface CPE can also reduce the R_ct_ by accelerating the electron transfer rate due to its high electrocatalytic ability. Compared to all the electrodes, poly (glutamic acid)/ZnO NPs–CPE has the lowest R_ct_. Thus, the combination of ZnO NPs and poly (glutamic acid) results in reduced interfacial charge transfer resistance and rapid heterogeneous electron transfer kinetics due to an increase in electroactive sites and conductivity. Therefore, the result obtained from EIS supports those obtained from CV. Furthermore, the exchange current density (Jo) for the electrodes was calculated from the EIS data using the following Eq. (2) [50].

2 $$\mathbf{J}\mathbf{o}=\frac{\mathbf{R}\mathbf{T}}{\mathbf{n}{\mathbf{R}}_{\mathbf{c}\mathbf{t}}\mathbf{A}\mathbf{F}}$$

where Jo is the exchange current density (A/cm ^2^), R the universal gas constant (8.314 J K^−1^ mol^−1^), T the temperature (298 K), F Faraday constant (96,485 C mol^−1^), R_ct_ is the electron transfer resistance (Ω), A is the electrode surface area (cm^2^) and n is the number of electrons involved in the reaction (n = 1). The Jo values obtained for the BCPE, ZnO NPs–CPE, poly (glutamic acid)/CPE and poly (glutamic acid)/ZnO NPs–CPE are 170, 401, 1760 and 5300 µA/cm^2^, respectively. The results showed a higher Jo value is obtained for the composite modified electrode due to high surface area and the existence of more active functional groups on the electrode surface which facilitate the electron transfer between the electrode and [Fe(CN)_6_]^3–/4–^. This result further confirms the composite modified electrode possessed good electrocatalytic activity.

### Crystal structure of ZnO NPs and surface morphology of the prepared electrodes

**Fig. 5:**
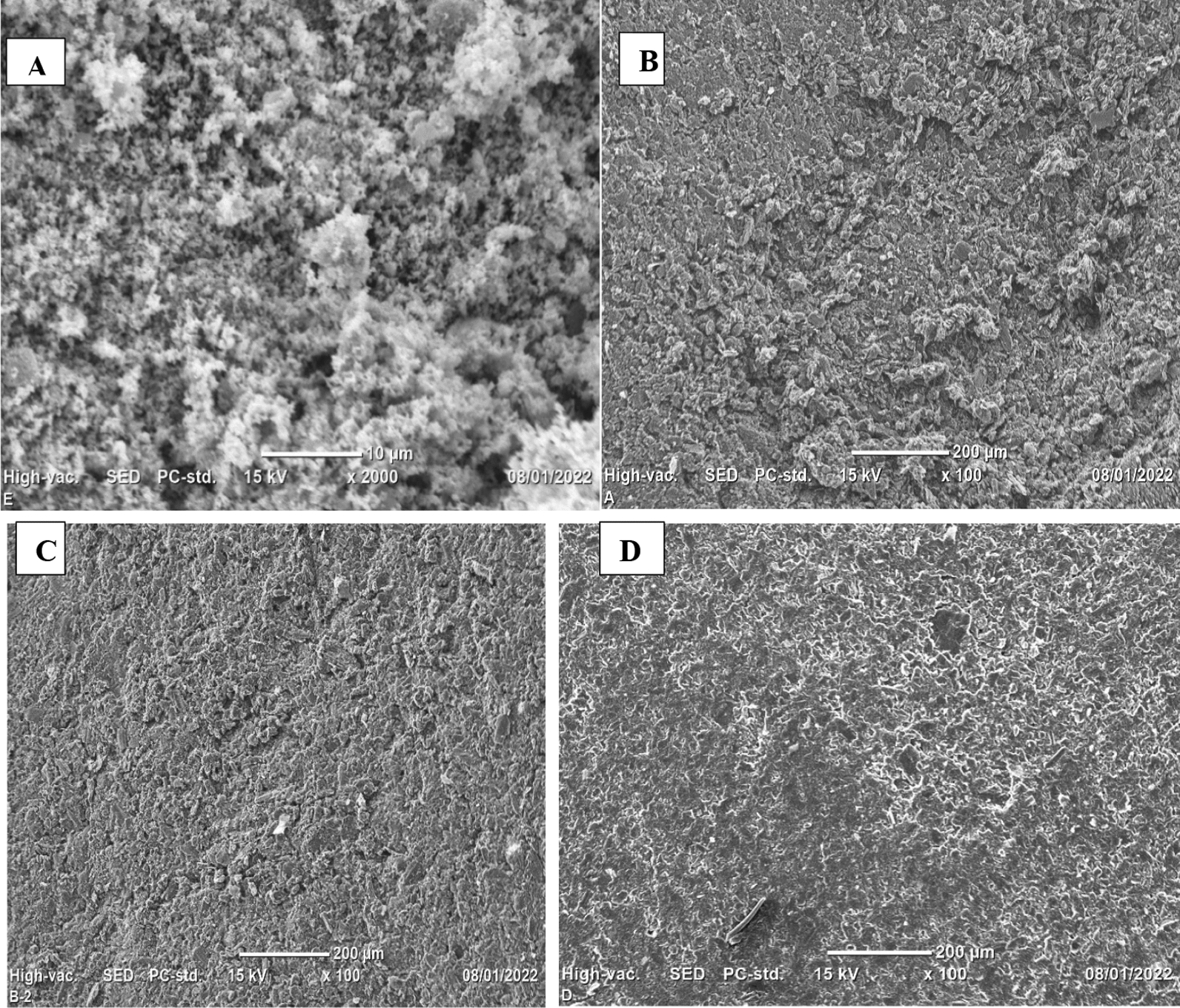
SEM images of **A** ZnO NPs, **B** BCPE, **C** ZnO NPs–CPE and **D** Poly (glutamic acid)/ZnO NPs–CPE

The surface morphologies of ZnO NPs, BCPE, ZnO NPs–CPE and poly (glutamic acid)/ZnO NPs–CPE were studied by SEM. Figure 5A shows ZnO NPs have non-uniform size distribution and irregular shape due to the agglomeration of the nanoparticles [51]. The BCPE surface has an irregularly shaped graphite layer and rough morphology (Fig. 5B). But, the SEM image of ZnO NPs–CPE showed ZnO NPs are uniformly distributed in the carbon paste, indicating the ZnO NPs were successfully incorporated in the CPE (Fig. 5C). Figure 5D shows that poly (glutamic acid) was uniformly and homogeneously deposited onto ZnO NPs–CPE surface, indicating the polymer film was successfully adhered to ZnO NPs–CPE surface.

**Fig.6:**
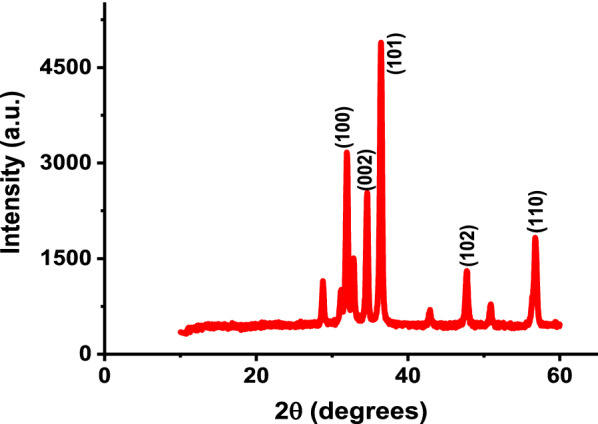
XRD pattern of ZnO NPs

XRD was used to determine the crystalline structure and grain sizes of the synthesized ZnO NPs. The intensity data were obtained over a 2θ range of 10–60°. As shown in Fig. 6, the strongest diffraction peaks observed at 2θ values of 31.92°, 34.62°, 36.44°, 47.74° and 56.76° have been indexed as (100), (002), (101), (102) and (110) crystal planes of ZnO, respectively. These peaks correspond to the hexagonal wurtzite structure of ZnO which is in agreement with the value in the standard card [52, 53]. The XRD peaks are found to be very sharp indicating good crystallinity of the sample grains. In addition to the above peaks, diffraction peaks with low intensities located near 28° and 42° were observed which can be indexed to the ZnO phase [53]. The XRD pattern indicates the presence of two crystalline phases of ZnO with low percentage in the latter phase [53]. The crystalline size of the synthesized ZnO NPs was calculated using Debye–Scherrer formula (Eq. 3) [52]

3 $$\mathbf{D}= \frac{{\varvec{K}}{\varvec{\lambda}}}{{\varvec{\beta}}{\varvec{C}}{\varvec{O}}{\varvec{S}}{\varvec{\theta}}}$$

where D is the crystalline size, K is a Scherrer’s constant of 0.94, λ is the wavelength of x-rays (λ = 1.542 Å), β is the full width at half maximum (FWHM) of the line, and θ is the Bragg diffraction angle. The grain size of the ZnO NPs was found to be 31 nm which was calculated using the most intense peak (2θ = 36.44°).

### Electrochemical behavior of vitamin B2 at bare CPE and modified CPEs

**Fig. 7:**
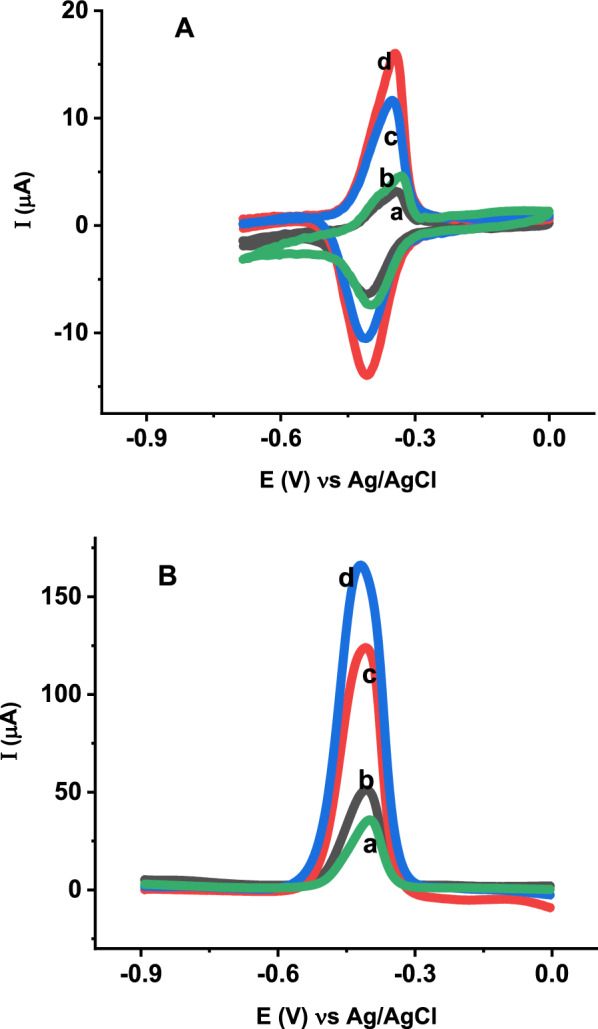
**A** CVs of **a** BCPE, **b** ZnO NPs–CPE, **c** Poly (glutamic acid)/CPE and **d** Poly (glutamic acid)/ZnO NPs–CPE at scan rate of 100 mV/s and **B** SWVs of **a** BCPE, **b** ZnO/CPE, **c** Poly (glutamic acid)/CPE and **d** Poly (glutamic acid)/ZnO NPs–CPE in the presence of 7.5 µM vitamin B2 in 0.1 M PBS pH 6. Conditions: open circuit accumulation for 180 s. Potential scan -1.0 V to 0.0 V. SWV parameters: frequency, 15 Hz; step potential, 4 mV and pulse amplitude, 40 mV

The voltammetric response of vitamin B2 was initially studied by CV at the surface of BCPE and modified electrodes at a scan rate of 100 mV/s in PBS pH 6. Figure 7A shows the CVs of 7.5 µM vitamin B2 at CPE (curve a), ZnO NPs–CPE (curve b), Poly (glutamic acid)/CPE (curve c) and poly (glutamic acid)/ZnO NPs–CPE (curve d) in PBS pH 6 at a scan rate of 100 mV/s. At the BCPE (curve a), vitamin B2 shows a quas- reversible redox behavior with weak redox peaks and peak-to-peak separation of 0.069 V. Similarly, a pair of quasi-reversible redox peaks slightly higher than that of bare CPE with peak-to-peak separation of 0.066 V was observed at ZnO NPs–CPE (curve b). A slight increase in peak current and decrease in pea-to-peak separation at ZnO NPs–CPE indicates the high electrocatalytic activity of ZnO NPs. At Poly (glutamic acid)/CPE redox peaks higher than that of the bare CPE and ZnO NPs–CPE were observed with peak potential separation of 0.062 V. The significantly increased redox peak currents were observed at poly (glutamic acid)/ZnO NPs–CPE due to the synergistic effect of ZnO NPs and Poly (glutamic acid) such as high conductivity, large effective surface area and presence of functional groups which facilitate accumulation and electron transfer rate of vitamin B2. Furthermore, the electrochemical behavior of vitamin B2 was investigated by SWV. As shown in Fig. 7B, the oxidation peak current produced at poly (glutamic acid)/ZnO NPs–CPE was higher than that of bare CPE, ZnO NPs–CPE and Poly (glutamic acid)/CPE.

## Optimization of experimental conditions

### Effect of amount of ZnO NPs on the electrooxidation of vitamin B2

**Fig. 8:**
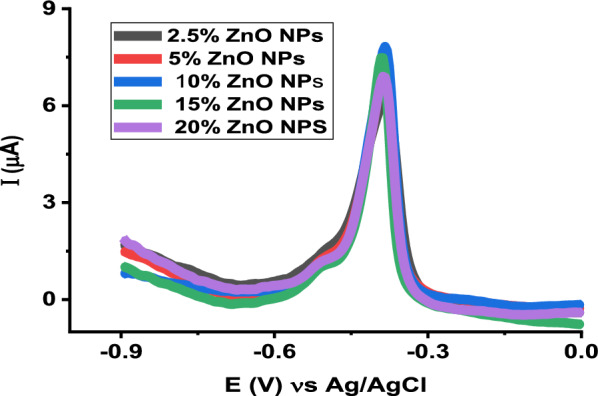
SWVs of 5 µM Vitamin B2 at the surface of the modified electrode with various amounts of zinc oxide nanoparticles (ZnO NPs)

The effect of the amount of ZnO NPs in the carbon paste on the current response of vitamin B2 was studied by SWV for 5 µM vitamin B2 in the range of 2.5–20% (w/w) with respect to graphite powder. As shown in Fig. 8, the oxidation peak current of vitamin B2 increased with increase in percentage up to 10% and thereafter, the peak current slightly decreased. This is because the active sites for adsorption of vitamin B2 increased with the increase in ZnO NPs percentage in the carbon paste, while the excess of ZnO NPs decreases the conductivity of the electrode. Thus, 10% of ZnO NPs was selected as the optimum amount for the preparation of poly (glutamic acid)/ZnO NPs–CPE.

### Optimization of electropolymerization parameters

In order to obtain the optimum polymerization conditions for glutamic acid, the effects of the electropolymerization parameters such as concentration of glutamic acid, number of cycles of polymerization, scan rate for polymerization and pH of supporting electrolyte for polymerization were investigated by measuring the current response of vitamin B2. Initially, different concentrations of glutamic acid were used for electropolymerization. As shown in Additional file 1: Fig. S2A, the current response of vitamin B2 increased with an increase in the concentration of glutamic acid up to 0.1 M then the current slightly decreased when the concentration of glutamic acid exceeded 0.1 M. This may be due to higher coverage of poly (glutamic acid) film on the electrode surface resulting in the decrease of electron transfer rate at the electrode surface [28, 35]. Next, the effect of the number of cycles of electroplymerization on the performance of the electrode was examined from 3 to 20 cycles in the presence of 0.1 M glutamic acid in 0.1 M PBS pH 7at a scan rate of 100 mV/s. As the number of cycles increased, the sensitivity of vitamin B2 also increased up to 7 cycles due to increase in active sites at the electrode surface (Additional file 1: Fig. S2B). The decrease in peak current after 7 cycles is due to increase in film thickness which blocks the electron transfer between the electrode and vitamin B2 []. Therefore, 7 potential scans were selected for the electropolymerization of glutamic acid on the electrode surface. The effect of pH of 0.1 M PBS on electropolymerizaton was investigated in the range 5 to 8. The highest sensitivity was obtained for vitamin B2 when 0.1 M PBS pH 7 was used for the electropolymerization of glutamic acid (Additional file 1: Fig. S2C). Finally, the effect of scan rate used for the electrodeposition of poly (glutamic acid) on the sensitivity of vitamin B2 was studied in the range from 25–150 mV/s. The highest current response was obtained with the scan rate of 50 mV/s (Additional file 1: Fig. S2D). Therefore, this scan rate was selected for the electropolymerization.

### Effect of supporting electrolyte and pH

**Fig. 9:**
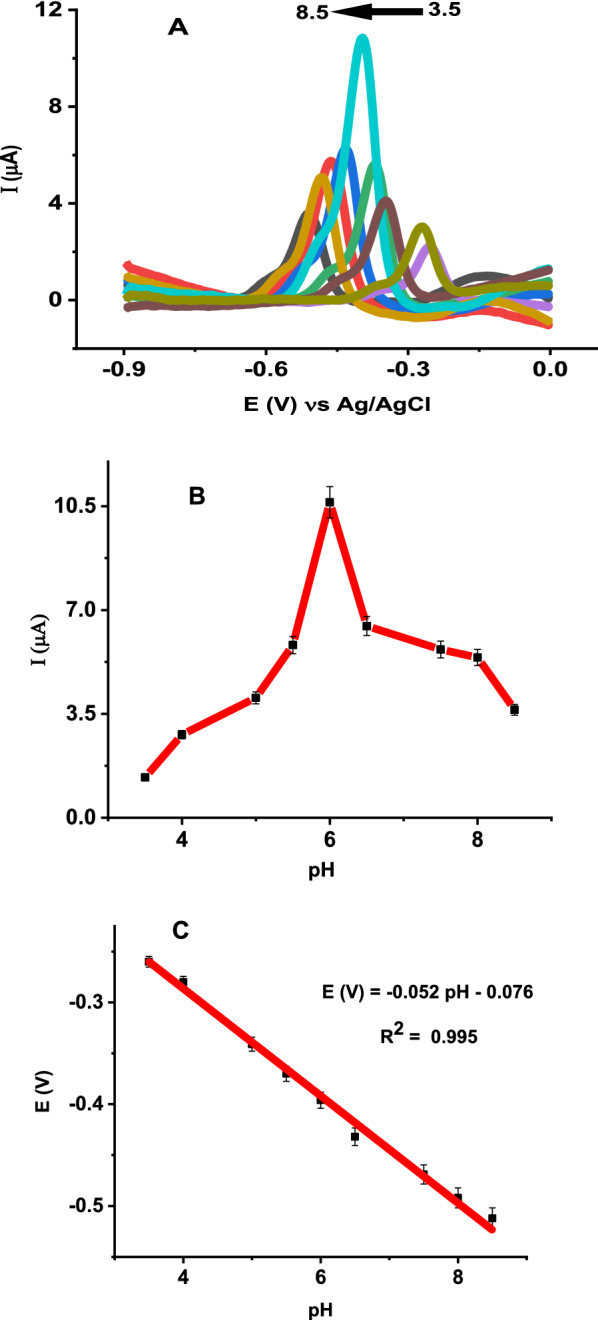
**A** SWVs of 5 µM vitamin B2 at poly (glutamic acid)/ ZnO NPs–CPE in 0.1 M PBS of various pH values (3.5, 4, 5, 5.5, 6, 6.5, 7.5, 8 and 8.5), **B** Effect of pH on the current responses of vitamin B2 and **C** Plots of peak potential of vitamin B2 versus the pH of buffer solution

The effect of different electrolytes including citrate buffer solution (CBS), phosphate buffer solution (PBS), acetate buffer solution (ABS), citrate–phosphate buffer solution (CB-PBS), Britton-Robinson buffer (BRB), HClO_4_ and KCl on the electrochemical response of 5 µM vitamin B2 at poly (glutamic acid)/ ZnO NPs–CPE were studied by SWV. As shown in Additional file 1: Fig. S3, a high peak current and a good peak shape were observed in 0.1 M PBS. Hence, 0.1 M PBS was selected as the supporting electrolyte for the determination of vitamin B2. The influence of the pH of PBS on the sensitivity of 5 µM vitamin B2 at poly (glutamic acid)/ ZnO NPs–CPE was studied in the pH range from 3.5 to 8 by SWV (Fig. 9A). As shown in Fig. 9B, the peak current of vitamin B2 increased gradually with increasing pH from 3.5 to 6 and then decreased gradually with increasing pH. The decrease in peak current at low pH and high pH can be explained as follows: at low pH, there is electrostatic repulsion between protonated poly (glutamic acid) and protonated vitamin B2 leading to a decrease in the accumulation of vitamin B2 at the electrode surface. Similarly, at high pH, the carboxylic functional group of poly (glutamic acid) is deprotonated leading to a decrease in electrostatic interaction between electron-rich amino group of vitamin B2 and negatively charged poly (glutamic acid). Furthermore, vitamin B2 is unstable and converted to lumichrome and lumiflavin in acidic and basic solutions, respectively [54]. Thus, 0.1 M PBS pH 6 was selected as the optimum value for the determination of vitamin B2 due to effective interaction between the electrode and vitamin B2 resulting in high sensitivity. Furthermore, the plot of peak potential of vitamin B2 versus pH of the solution at poly (glutamic acid)/ZnO NPs–CPE indicates the dependence of peak potential on pH. Figure 9C shows peak potential varies linearly with pH with the equation: E_p_ (V) =  −  0.052 pH − 0.076 (R^2^ = 0.995). The slope is close to the Nernstian value of −  59 mV, indicating that the electrochemical oxidation of vitamin B2 involves an equal number of protons and electrons, which is in agreement with previous works [7, 55 − 55].

### Effect of scan rate

**Fig. 10:**
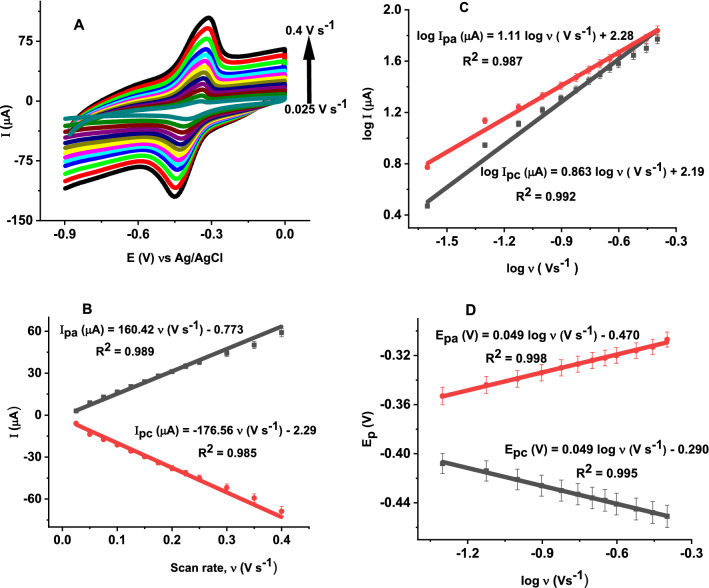
**A** CVs of 5 µM vitamin B2 at poly (glutamic acid)/ZnO NPs–CPE in PBS pH 6 at scan rates of 0.025, 0.05, 0.075, 0.1, 0.125, 0.15, 0.175, 0.2, 0.225, 0.25, 0.3, 0.350, 0.4 V s.^−1^, **B** Plot of **a** Anodic and **b** Cathodic peak currents versus scan rate (υ), **C** Variation of the logarithm of **a** Anodic and **b** Cathodic peak currents (log I) with logarithm of scan rate (log υ) and **D** Variation of **a** Anodic and **b** Cathodic peak potentials (Ep) with logarithm of scan rate (logυ)

To investigate the mechanism involved in the electrochemical process, the effect of scan rate on the peak current and peak potential of 5 µM vitamin B2 in PBS pH 6 at the poly (glutamic acid)/ZnO NPs–CPE was studied by cyclic voltammetry. Figure 10A shows cyclic voltammogram of 5 µM vitamin B2 at different scan rates in the range 0.025 to 0.4 V s^−1^. As shown in Fig. 10B, both anodic the and cathodic peak currents increased linearly with scan rate with regression equations: I_pa_ (µA) = 160.42 υ (Vs^−1^) – 0.773 (R^2^ = 0.989) and I_pc_ (µA) =–176.56 − 2.29 υ (Vs^−1^) (R^2^ = 0.985). These results suggest the electrode reaction is controlled by adsorption of vitamin B2 which is in consistence with previous reports [7, 11, 55]. On the other hand, there exists a good linear relationship between the logarithm of peak current and the logarithm of scan rate in the range 0.025 to 0.4 Vs^−1^ with regression equations: log I_pa_ (µA) = 1.11 log υ (Vs^−1^) + 2.28 (R^2^ = 0.985) for the oxidation and log I_pc_ (µA) = 0.863 log υ (Vs^−1^) + 2.19 (R^2^ = 0.992) for the reduction reactions (Fig. 10C). The slopes values are 1.11 and 0.863 for the oxidation and reduction peak currents, respectively. These values are close to the theoretical value of 1 which further confirms the electrochemical reaction of vitamin B2 at poly (glutamic acid)/ZnO NPs–CPE is a surface-controlled electrode process [7, 11, 58]. Furthermore, the influence of scan rate on the redox peak potential was investigated. With the increase in scan rate, the oxidation and reduction peak potentials shifted slightly in the positive and negative direction, respectively, indicating the electrode reaction is quasi-reversible [55]. The electron transfer coefficient (α) and the number of electrons (n) were calculated using Laviron’s equation for quasi-reversible systems [55, 57].

4 $$\mathrm{E_{pa} }=\mathrm{ E^o}+\frac{2.303\mathrm{RT}}{(1-\mathrm{\alpha })\mathrm{nF}} \left(\mathrm{log \upsilon }+\mathrm{log}\frac{\left(1-\mathrm{\alpha }\right)\mathrm{nF}}{\mathrm{RTks}}\right)$$

5 $$\mathrm{E_{pc} }=\mathrm{ E^o}-\frac{2.303\mathrm{RT}}{\mathrm{\alpha nF}}\left(\mathrm{log \upsilon }+\mathrm{log }\frac{\mathrm{\alpha nF}}{\mathrm{RTks }}\right)$$

where E^o^ is the formal potential, T is the temperature (298 K), α is the transfer coefficient, n is the number of electrons transferred, υ is the scan rate, F is the Faraday’s constant (96,480 C mol^−1^), R is the universal gas constant (8.314 J mol^−1^ K^−1^), k_s_ is the heterogeneous electron-transfer rate constant and ∆Ep is the peak-to-peak potential separation.

The plots of the oxidation and reduction peak potentials versus the logarithm of scan rate (log υ) in the range 0.075 to 0.4 Vs^−1^ are shown in Fig. 10D. The corresponding regression equations are: E_pa_ (V) = 0.049 log υ (Vs^−1^) – 0.470 (R^2^ = 0.998) and E_pc_ (V) =  − 0.049 log υ (Vs^−1^) − 0.290 (R^2^ = 0.995) for the oxidation and reduction reactions, respectively. According to Laviron’s equation (Eq. 4 and 5), the slops are equal to 2.3RT/ (1 − α) nF and − 2.3RT/αnF for the anodic and the cathodic peaks, respectively. Thus, the electron transfer coefficient (α) and the number of electrons transferred (n) were calculated to be 0.5 and 2.34 (~ 2), respectively. Furthermore, the heterogeneous electron transfer rate constant (k^o^) was calculated using (Eq. 6) [7].

6 $$\Delta {\text{E}} = 201.39{\text{ log}}\,( \upsilon /{\text{k}}^{\circ} ) - 301.78$$

where k^o^ is the heterogeneous electron-transfer rate constant. The values of ΔEp and the respective values of k^o^ for the oxidation of vitamin B2 are listed in Additional file 1: Table S1. The result indicates k^o^ increased with the increase in scan rate due to the availability of more active sites on the electrode surface [7]. The average value of k^o^ was found to be 1.77 s^−1^. This value is higher than those reported previously at GCE modified with reduced graphene oxide (0.082 s^−1^) and GCE modified with functionalized carbon nanotube (0.66 s^−1^)[57]. This relatively high value of k^o^ indicates the poly (glutamic acid) and ZnO NPs composite can effectively enhance the electron transfer between the electrode surface and vitamin B2. Since the electrode reaction for vitamin B2 is adsorption—controlled process, its adsorption capacity at poly (glutamic acid)/ZnO NPs–CPE surface was calculated using Eq. (7) [59].

7 $$\mathbf{Ipa} =\frac{{\mathbf{n}}^{\mathbf{2}}{\mathbf{F}}^{\mathbf{2}}\Gamma \mathbf{A}{\varvec{\upupsilon}}}{\mathbf{4RT}}$$

where Γ is the absorption capacity (mol cm^−2^), υ is the scan rate, and A, R, T, F and n are as described in Eq. (2). The surface coverage of vitamin B2 at the poly (glutamic acid)/ZnO NPS–CPE was calculated to be Ґ = 4.1 × 10^–4^ mol cm^−2^.

**Scheme 3:**
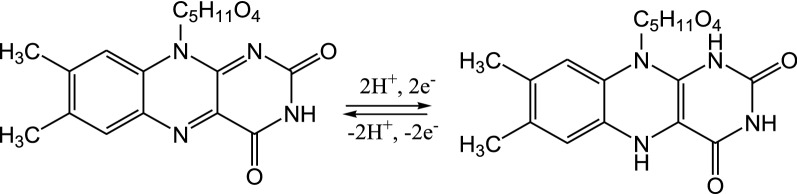
Mechanism for the electrochemical reaction of vitamin B2

From the pH study, the number of electrons and protons that participated during the electrochemical reaction of vitamin B2 are equal. In addition, the scan rate study indicates two electrons are involved in the oxidation of vitamin B2. Therefore, the electrochemical reaction for vitamin B2 oxidation at poly (glutamic acid)/ ZnO NPs–CPE can be summarized as in Scheme 3.

### Effect of accumulation potential and time

The influence of accumulation potential (E_acc_) on the oxidation peak currents for 5 µM vitamin B2 in PBS pH 6 was studied using SWV in the range −  0.8 to 0.8 V and at accumulation time (t_acc_) of 60 s. With the change of accumulation potential, the peak current of vitamin B2 varied slightly, indicating the accumulation potential had an insignificant effect on the peak current of vitamin B2 at poly (glutamic acid)/ZnO NPs–CPE. Therefore, accumulation was carried out at open-circuit conditions. Furthermore, the effect of t_acc_ on the oxidation peak current for 5 µM vitamin B2 was also investigated in the range of 30 to 300 s. As shown in Additional file 1: Fig. S4, the peak current increased with increasing t_acc_ up to 180 s and then decreased due to surface saturation of the electrode. Considering sensitivity and analysis time, t_acc_ of 180 s was selected as the optimum accumulation time for determination of vitamin B2.

### Effect of SWV parameters for the determination of vitamin B2

SWV parameters such as step potential, pulse amplitude and frequency influence the current response and the shape of SW voltammogram. The effect of frequency on the current responses for 7.5 µM vitamin B2 in PBS pH 6 was studied in the range 5–60 Hz by fixing step potential constant at 4 mV and amplitude at 25 mV. The influence of step potential was investigated in the range 2–18 mV by fixing the frequency at 15 Hz and the amplitude at 25 mV. Finally, the amplitude was also optimized in the range 10–90 mV by keeping the frequency and the step potential at 15 Hz and 4 mV, respectively. The optimum values obtained are 15 Hz, 4 mV and 40 mV for the frequency, step potential and amplitude, respectively.

### Determination of vitamin B2 at poly (glutamic acid)/ZnO NPs–CPE

**Fig. 11:**
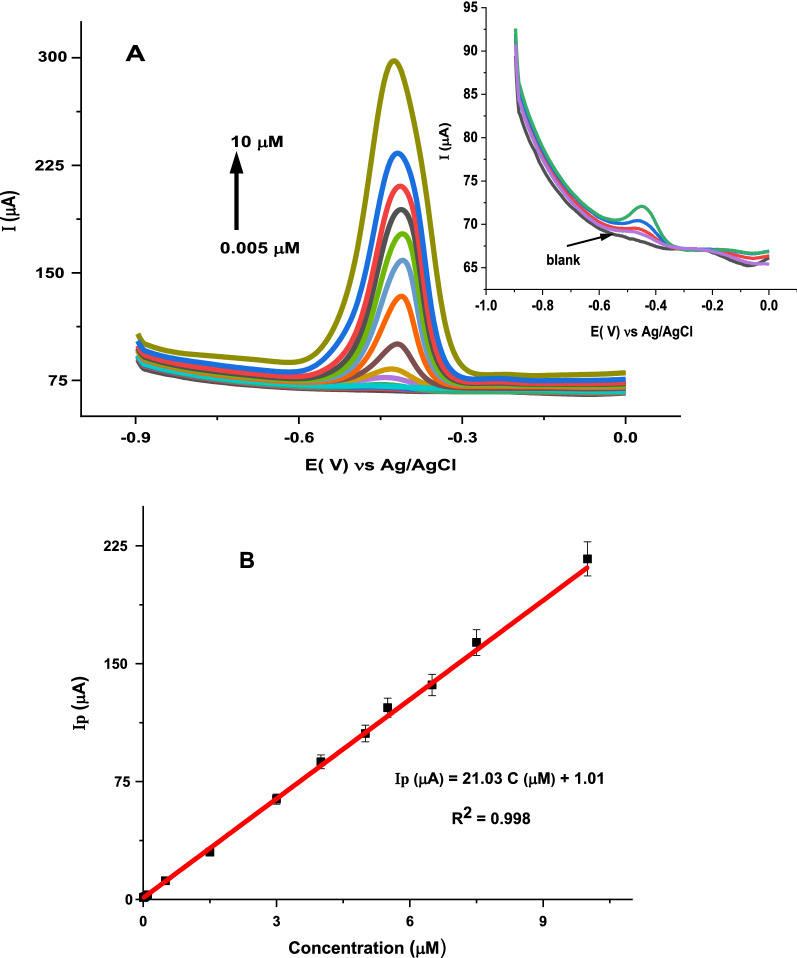
**A** SWVs for different concentrations of vitamin B2: 0.005, 0.02, 0.04, 0.1, 0.5, 1.5, 3, 4, 5, 5.5, 6.5, 7.5 and 10 µM in 0.1 M PBS pH 6 at poly (glutamic acid)/ZnO NPs–CPE: Inset: magnified SWVs for lower concentrations (0.005–0.04 µM) and **B** Plot of the peak current versus concentration of vitamin B2 at poly (glutamic acid)/ZnO NPs–CPE. Conditions: open circuit accumulation for 180 s. Potential scan -1.0 V to 0.0 V. SWV parameters: frequency, 15 Hz; step potential, 4 mV and pulse amplitude, 40 mV

Under the optimized experimental parameters, SWV was used for the determination of vitamin B2 in PBS pH 6.0 in the potential range − 1.0 to 0.0 V. As shown in Fig. 11A, the peak currents of vitamin B2 increased upon increasing the concentrations and a linear response was obtained in the range 0.005–10 µM. As shown in the Fig. 11B, the regression equation is: I (µM) = 21.03 C (µM) + 1.01, R^2^ = 0.996. The values of the LOD and limit of quantification (LOQ) were calculated using the equations LOD = 3 s⁄m and LOQ = 10 s/m, where s represents the standard deviation of the peak current (n = 6) of the lowest concentration in the linear range and m is the slope of the calibration curve. The LOD and LOQ values calculated are 0.0007 ± 0.00001 µM and 0.0023 ± 0.00006 µM, respectively.

The comparison of the analytical performance of the proposed method with previous reported electrochemical methods for the determination of vitamin B2 is given in Table 1. The results indicate the proposed sensor shows a comparable linear range with most of the reported sensors and provides a lower detection limit for the analysis of vitamin B2 at sufficiently low concentration. Furthermore, our proposed method provides several advantages such as simple to prepare, low cost and quick response during analysis.

**Table 1:** Comparison of different electrochemical sensors reported for the determination of vitamin B2

Modified electrode	Technique	Linear range (µM)	LOD (µM)	References
ERGO/GCE	DPV	0.1–40	0.016	[57]
AuNPs@PDA-RGO/GCE	DPV	0.02–60	0.0096	[55]
α-Fe_2_O_3_/ MWCNT/AuNPs/GCE	SWV	0.3–60	0.006	[46]
PNN/GPE	LSV	5–60	0.782	[11]
MnTPP/CPE	DPV	0.01–10	0.008	[6]
P_3_MT/GCE	DPV	0.1–200	0.05	[8]
PDLPAMCPS	DPV	6–30	0.092	[7]
MnO_2_/GCE	DPV	2–110	0.027	[60]
MnO_2_/CPE	DPV	0.02–9	0.015	[61]
GF/GCE	DPV	0.001–0.015	0.0001	[58]
PAMCPE	DPV	2–50	0.093	[12]
MB-SO_3_H-MSM/GCE	DPV	0.01–50	0.005	[56]
poly (glutamic acid)/ZnO NPs- CPE	SWV	0.005–10	0.0007	This work

*ERGO* electrochemically reduced graphene oxide, *AuNPs@PDA–RGO/GCE* gold nanoparticles on glassy carbon electrode modified by a hybrid of polydopamine and reduced graphene oxide; MWCNT = multi- walled carbon nanotube, *PNNMGPE* poly (niacin) modified graphite paste electrode; LSV = linear sweep voltammetrym, *MnTPP/CPE* manganese (III) tetraphenylporphyrin modified carbon paste electrode, *P*_*3*_*MT/GCE* poly (3–methylthiophene) modified glassy carbon electrode, *PDLPAMCPS* poly (DL-phenylalanine) modified carbon sensor; *MnO2/GCE* manganese dioxide modified glassy carbon electrode, *GF-GCE* graphene film modified glassy carbon electrode; PAMCPE─Poly(arginine) modified carbon paste electrode, *MB-SO*_*3*_*H-MSM* methylene blue incorporated mesoporous silica microsphere, *DPV* Differential pulse voltammetry

### Repeatability, reproducibility and stability of poly (glutamic acid)/ZnO NPs–CPE

The repeatability of the modified electrode was evaluated by ten successive measurements of 5 µM vitamin B2. The results showed a relative standard deviation (RSD) of 1.2% was obtained, which indicates the fabricated sensor has good repeatability for the determination of vitamin B2. To investigate the reproducibility of the modified electrode, six different electrodes were prepared under the same conditions and SWVs were recorded for 5 µM vitamin B2. RSD of 2.25% was obtained, indicating excellent reproducibility of the responses at poly (glutamic acid)/ZnO NPs ─CPE. In addition, the stability of the electrode was investigated by measuring the peak current signals of 5 µM vitamin B2 after one-month of storage of the electrode at 4 ^◦^C in a refrigerator. At the end of the month, the oxidation current response decreased by 5.08%, indicating good stability of the prepared electrode.

### Interference study

The selectivity of poly (glutamic acid)/ZnO NPs─CPE for the determination of 3.75 μM vitamin B2 was investigated by SWV in the presence of interfering substances that commonly coexist with vitamin B2 in non-alcoholic beverages and milk samples. The oxidation peak currents of vitamin B2 obtained in the presence of interfering substances were compared with those obtained in the absence of each interferent. As shown in Additional file 1: Table S2, 100-fold excess concentrations of Ca^2+^, Na^+^, Mg^2+^, Cu^2+^, Fe^2+^, NO_3_^−^_,_ CO_3_^2−^ and HCO_3_^2−^, 50–fold excess concentrations of ascorbic acid, citric acid, glucose, starch, sucrose and lactose and 25 -fold excess concentration of vitamin B1, vitamin B6, vitamin B9 and vitamin B12 had no effect on the voltammetric responses of vitamin B2. The percent changes in the peak current response are less than 5%, indicating the presence of these possible interfering species had insignificant effect on the peak currents of vitamin B2. Therefore, the proposed electrode has high selectivity for the determination of vitamin B2 in beverage and milk samples.

### Analytical application

The application of the developed method for real samples analysis was examined in the determination of vitamin B2 in non-alcoholic beverages (malt guinness) and milk (nido milk) samples. The samples were prepared according to the procedure described in Sect. 2.3 and diluted to concentrations within the range of the linear calibration curve. Standard addition method was applied for quantification of vitamin B2 in malt Guinness and milk samples and the results are shown in Table 2. Additional file 1: Fig. S5 shows the square-wave voltammograms for the sample alone and after consecutive additions of standard solutions of vitamin B2. As can be seen from the voltammograms, for unspiked samples a peak was observed for vitamin B2 at the same potentials as that observed in the case of standard vitamin B2 and its peak current increased upon consecutive addition of vitamin B2 standard solutions, which indicates both samples contain vitamin B2.

**Table 2:** Recovery study

Sample	Added (µM)	Found (µM) ± RSD (%) n = 3	Recovery (%)
Malt Guinness	0	0.51 ± 3.01	–
	0.2	± 0.78	94
	0.5	0.95 ± 1.8	88
	1	1.41 ± 0.52	90
	2	2.41 ± 1.15	95
	2.5	2.83 ± 1.63	92.8
Nido milk	0	0.39 ± 1.5	–
	0.2	0.57 ± 1.04	90
	0.5	0.85 ± 5.8	92
	1	1.33 ± 3.5	94
	2	2.4 ± 2.2	100.5
	2.5	2.9 ± 1.5	100.4

Furthermore, in order to evaluate the applicability of the proposed method, recovery experiments were performed by spiking the samples with different concentrations of vitamin B2 standard solutions. As shown in Table 2, acceptable recoveries were obtained ranging from 88% to 100.5%. These recovery values indicate that there are no significant interferences from the matrices in the sample. Furthermore, the percent relative standard deviation (RSD %) values varied from 0.52 to 5.8%, suggesting good precision of the measurements. The accuracy of the method was also evaluated by using the t-test. The calculated value of t (t_cal_) using Eq. 8 was found to be 1.15 and 4.1 for malt guinness and nido milk, respectively (Table 3). At degrees of freedom 2 (n − 1), the critical value is t_2_ = 4.3 (P = 0.05). Therefore, the critical t- value is greater than the calculated t- value (t_cal_) at 95%. Thus, the null hypothesis is retained or there is no significance difference between labelled and measured results. Therefore, the modified electrode can be used for the determination of vitamin B2 in non-alcoholic beverages and milk samples with high accuracy, precision and selectivity.

8 $$\frac{(\mathbf{X}-{\varvec{\upmu}})\sqrt{\mathbf{n}}}{\mathbf{S}}$$

where X is sample mean, µ is the labelled value, S is the sample standard deviation and n is number of samples.

**Table 3:** Comparison of experimental results with labelled values

Sample	Labelled	Experimental ± SD	Relative error (%)	t_cal_
Malt Guinness	0.18 mg/100 ml	0.19 mg/100 ml ± 0.015	5.1	1.15
Nido milk	1.4 mg/100 g	1.46 mg/100 g ± 0.025	4.3	4.1

*SD* Standard deviation for three replicate measurements. t_2_ = 4.3 (P = 0.05)

## Conclusion

In summary, a highly sensitive electrochemical sensor was successfully prepared from poly (glutamic acid) and ZnO NPs composite for the determination of vitamin B2. The fabricated sensor has high electrocatalytic activity towards the electrochemical oxidation of vitamin B2. The high sensitivity and lower detection limit at the composite modified electrode are due to high conductivity, large effective surface area and strong accumulation ability of the electrode through hydrogen bonding and π-π interactions between the polymer film and vitamin B2. Furthermore, the combined effect of ZnO NPs and poly (glutamic acid) improved the standard heterogeneous rate constant (k^o^), which indicates a relatively fast electrode reaction process. Under the optimized experimental conditions, the developed sensor exhibited a wide linear range (0.005─10 µM) and low LOD (0.0007 µM) for the determination of vitamin B2. The acceptable recovery values indicate the proposed method can be successfully used for the determination of vitamin B2 in non-alcoholic beverage and milk samples with high accuracy and selectivity. In addition, the proposed sensor has significant advantages, such as easy to prepare, cost-effectiveness, fast response and long-term stability.

## Supplementary Information

**Additional file 1:** **Fig. S1.** Plot of peak current versus square root of scan rate for **a** BCPE, **b** ZnO−CPE, **c** Poly (glutamic acid)/CPE and **d** poly (glutamic acid)/ ZnO NPs−CPE. **Fig. S2.** Effect of **A** Concentration of glutamic acid, **B** Polymerization cycles, **C** pH and **D** Scan rate on the oxidation current of vitamin B2. **Fig. S3** SWVs of 5 µM vitamin B2 at poly (glutamic acid)/ ZnO NPs−CPE in different supporting electrolyte. **Fig. S4. **Effect of t_acc_ on the oxidation peak currents of 5 µM vitamin B2. **Table S1. **Heterogeneous rate constants of vitamin B2 at poly (glutamic acid) /ZnO NPs- CPE. **Table S2.** Influence of potential interferants on the voltammetric response of 3.75 μM vitamin B2 at poly (glutamic acid)/ZnO NPs−CPE (n=3). **Fig. S5. **SWVs recorded at poly (glutamic acid)/ZnO NPs−CPE in phosphate buffer solution pH 6.0 (background subtracted) for **A** Malt gunnesse sample and **B** milk sample.

## Data Availability

The data sets generated or analyzed during the current study are available to readers as in the manuscript and Additional file 1. The data obtained for CV, EIS, SEM and XRD during electrode characterization have been deposited in figshare (https://doi.org/10.6084/m9.figshare.19603363, https://doi.org/10.6084/m9.figshare.19603798, https://doi.org/10.6084/m9.figshare.19603825, and https://doi.org/10.6084/m9.figshare.19603879, respectively).

## Abbreviations

ABS: Acetate buffer solution
BRB: Britton-Robinson buffer
CB-PBS: Citrate–phosphate buffer solution
CBS: Citrate buffer solution
CPE: Carbon paste electrode
CV: Cyclic voltammetry
EIS: Electrochemical impedance spectroscopy
LOD: Limit of detection
LOQ: Limit of quantification
PBS: Phosphate buffer solution
SEM: Scanning electron microscopy
SWV: Square wave voltammetry
XRD: X-Ray diffraction
ZnO NPs: Zinc oxide nanoparticles
