# Supplementary material for: Sensitive and selective determination of vitamin B2 in non-alcoholic beverage and milk samples at poly (glutamic acid)/zinc oxide nanoparticles modified carbon paste electrode
Source: BMC Chem. 2022 Sep 18;16(1):69. doi: 10.1186/s13065-022-00863-5 (PMC9482753; doi:10.1186/s13065-022-00863-5)
Supplement: Supplementary file 1 — Additional file 1: Fig. S1. Plot of peak current versus square root of scan rate for a BCPE, b ZnO−CPE, c Poly (glutamic acid)/CPE and d poly (glutamic acid)/ ZnO NPs−CPE. Fig. S2. Effect of A Concentration of glutamic acid, B Polymerization cycles, C pH and D Scan rate on the oxidation current of vitamin B2. Fig. S3 SWVs of 5 µM vitamin B2 at poly (glutamic acid)/ ZnO NPs−CPE in different supporting electrolyte. Fig. S4. Effect of tacc on the oxidation peak currents of 5 µM vitamin B2. Table S1. Heterogeneous rate constants of vitamin B2 at poly (glutamic acid) /ZnO NPs- CPE. Table S2. Influence of potential interferants on the voltammetric response of 3.75 μM vitamin B2 at poly (glutamic acid)/ZnO NPs−CPE (n=3). Fig. S5. SWVs recorded at poly (glutamic acid)/ZnO NPs−CPE in phosphate buffer solution pH 6.0 (background subtracted) for A Malt gunnesse sample and B milk sample. [file 13065_2022_863_MOESM1_ESM.docx]

**Fig. S1** Plot of peak current versus square root of scan rate for **a** BCPE, **b** ZnO−CPE, **c** Poly (glutamic acid) / CPE and **d** poly (glutamic acid)/ ZnO NPs−CPE

**Fig. S2** Effect of **A** Concentration of glutamic acid, **B** Polymerization cycles, **C** pH and **D** Scan rate on the oxidation current of vitamin B2

**Fig. S3** SWVs of 5 µM vitamin B2 at poly (glutamic acid)/ ZnO NPs−CPE in different supporting electrolyte

**Fig. S4** Effect of t_acc_ on the oxidation peak currents of 5 µM vitamin B2

**Table S1** Heterogeneous rate constants of vitamin B2 at poly (glutamic acid) /ZnO NPs- CPE

| υ (mV/s) | ∆E (mV) | k^o^ (s^-1^) |
| --- | --- | --- |
| 70 | 70 | 1.06 |
| 100 | 82 | 1.23 |
| 125 | 92 | 1.39 |
| 150 | 100 | 1.52 |
| 175 | 106 | 1.66 |
| 200 | 112 | 1.76 |
| 225 | 116 | 1.90 |
| 250 | 121 | 1.99 |
| 300 | 129 | 2.17 |
| 350 | 135 | 2.37 |
| 400 | 144 | 2.47 |

**Table** S2 Influence of potential interferants on the voltammetric response of 3.75 μM vitamin B2 at poly (glutamic acid)/ZnO NPs−CPE (n=3)

| Interferants | Concentration (µM) | Current (µA) | Signal change (%) |
| --- | --- | --- | --- |
| Vitamin B2 | 3.75 | 80.74 | - |
| Glucose | 187.5 | 76.90 | - 4.8 |
| Ascrobic acid | 187.5 | 81.19 | + 0.56 |
| Starch | 187.5 | 79.06 | - 2.08 |
| Lactose | 187.5 | 77.89 | - 3.53 |
| Sucrose | 187.5 | 79.95 | - 0.98 |
| Citric acid | 187.5 | 80.86 | + 0.15 |
| Vitamin B9 | 187.5 | 80.79 | + 0.06 |
| Vitamin B6 | 187.5 | 79.93 | -1.0 |
| Vitamin B12 | 187.5 | 80.21 | - 0.66 |
| Vitamin B1 | 187.5 | 78.58 | -2.68 |
| Fe^2+^ | 375 | 79.57 | - 1.45 |
| Na^+^ | 375 | 81.06 | + 0.40 |
| Ca^2+^ | 375 | 78.7 | - 2.53 |
| Mg^2+^ | 375 | 78.19 | - 3.16 |
| Cu^2+^ | 375 | 75.48 | - 6.5 |
| NO_3_^-^ | 375 | 79.57 | - 1.45 |
| CO_3_^2-^ | 375 | 81.06 | + 0.40 |
| HCO_3_^2-^ | 375 | 83.03 | + 2.84 |

**Fig. S5** SWVs recorded at poly (glutamic acid)/ZnO NPs−CPE in phosphate buffer solution pH 6.0 (background subtracted) for **A** Malt gunnesse sample and **B** milk sample. **a** Before spiking and after spiking with **b** 0.2 µM **c** 0.5 µM **d** 1 µM **e** 2 µM and **f** 2.5 µM vitamin B2 standard solution. Conditions: open circuit accumulation for 180 s. Potential scan -1.0 V to 0.0 V. SWV parameters: frequency, 15 Hz; step potential, 4 mV and pulse amplitude, 40 mV. Inset: standard addition curve for vitamin B2
